# High Acetate Concentration Protects Intestinal Barrier and Exerts Anti-Inflammatory Effects in Organoid-Derived Epithelial Monolayer Cultures from Patients with Ulcerative Colitis

**DOI:** 10.3390/ijms24010768

**Published:** 2023-01-01

**Authors:** Sara Deleu, Kaline Arnauts, Lowie Deprez, Kathleen Machiels, Marc Ferrante, Geert R. B. Huys, Johan M. Thevelein, Jeroen Raes, Séverine Vermeire

**Affiliations:** 1 Department of Chronic Diseases, Metabolism & Ageing (CHROMETA), KU Leuven, 3000 Leuven, Belgium; 2 Department of Development and Regeneration, Stem Cell Institute Leuven, KU Leuven, 3000 Leuven, Belgium; 3 Department of Gastroenterology and Hepatology, University Hospitals Leuven, KU Leuven, 3000 Leuven, Belgium; 4 Center for Microbiology, VIB, 3000 Leuven, Belgium; 5 Department of Microbiology and Immunology, Rega Institute, KU Leuven, 3000 Leuven, Belgium; 6 Laboratory of Molecular Cell Biology, Institute of Botany and Microbiology, KU Leuven, 3000 Leuven, Belgium

**Keywords:** probiotics, prebiotics, SCFA, short chain fatty acids, acetate, ulcerative colitis, IBD, inflammatory bowel disease

## Abstract

Short-chain fatty acids as well as their bacterial producers are of increasing interest in inflammatory bowel diseases. Although less studied compared to butyrate, acetate might also be of interest as it may be less toxic to epithelial cells, stimulate butyrate-producing bacteria by cross-feeding, and have anti-inflammatory and barrier-protective properties. Moreover, one of the causative factors of the probiotic potency of *Saccharomyces cerevisae* var. *boulardii* is thought to be its high acetate production. Therefore, the objective was to preclinically assess the effects of high acetate concentrations on inflammation and barrier integrity in organoid-based monolayer cultures from ulcerative colitis patients. Confluent organoid-derived colonic epithelial monolayers (*n* = 10) were exposed to basolateral inflammatory stimulation or control medium. After 24 h, high acetate or control medium was administered apically for an additional 48 h. Changes in TEER were measured after 48 h. Expression levels of barrier genes and inflammatory markers were determined by qPCR. Pro-inflammatory proteins in the supernatant were quantified using the MSD platform. Increased epithelial resistance was observed with high acetate administration in both inflamed and non-inflamed conditions, together with decreased expression levels of IL8 and TNFα and CLDN1. Upon high acetate administration to inflamed monolayers, upregulation of HIF1α, MUC2, and MKI67, and a decrease of the majority of pro-inflammatory cytokines was observed. In our patient-derived human epithelial cell culture model, a protective effect of high acetate administration on epithelial resistance, barrier gene expression, and inflammatory protein production was observed. These findings open up new possibilities for acetate-mediated management of barrier defects and inflammation in IBD.

## 1. Introduction

The human gut microbiome has been shown to play an important role in health and disease [1]. Several disorders, including inflammatory bowel diseases (IBD), have been linked to changes in the faecal microbiota composition [2]. For both Crohn’s disease (CD) and ulcerative colitis (UC), the two main entities of IBD, a decrease in α-diversity and a lower species richness, as well as an increase in the prevalence of the potentially dysbiotic *Bacteroides2* enterotype compared to healthy controls has been observed [3,4]. In terms of bacterial abundances, reduction of specific beneficial bacterial taxa such as *Bifidobacterium* spp. and *Clostridium* Groups IV and XIVa including *Faecalibacterium prausnitzii* and *Roseburia* spp. have been demonstrated in IBD patients [5,6]. Contrarily, a relative increase in bacteria possessing inflammatory properties such as *Pasteurellaceae*, *Escherichia coli*, and *Fusobacteriaceae* has been observed [6,7,8]. 

The potential option to treat or improve quality of life of IBD patients by modulating the gut microbiome composition is being studied intensively [9]. While faecal microbial transplantation studies have shown promising results in open label and placebo-controlled trials [10,11,12,13], success rates seem donor- and recipient-dependent [14]. The use of well-defined probiotics is an alternative option that does not rely on donor availability and deals with potential concerns regarding unwanted side effects. For instance, the yeast *Saccharomyces cerevisiae* var. *boulardii* (e.g., Enterol^®^) might be used as a supportive treatment to reduce relapses in IBD patients [15]. A recent randomised, double-blind, placebo-controlled trial observed decreased intestinal inflammation upon use of a multi-strain bacterial probiotic treatment [16]. 

Another potential therapeutic strategy is based on the observed alterations in faecal microbial metabolite concentrations [17], including a reduction in short-chain fatty acids (SCFA), dysregulation of bile acid derivatives, and tryptophan metabolites in IBD patients [3,17]. SCFA such as acetate and butyrate have therefore gained interest for their potential beneficial effects in IBD [18]. Most studies have focused on butyrate, and have shown beneficial effects on gut microbiome composition, intestinal barrier function, and inflammation [19]. However, in the tested concentration range of 3–8 mM, butyrate has also been shown to have a toxic effect on epithelial colon cells in vitro, especially in the presence of an altered mucus layer, which is often the case in UC [20]. Acetate’s mode-of-action is less well known, although its lower toxicity to epithelial cells, as well as its potential to support growth of butyrate-producing bacteria by metabolic cross-feeding make the compound interesting for modulation purposes [21]. Moreover, a recent report suggested that the probiotic potential of *Saccharomyces cerevisiae* var. *boulardii* might be related to its unusually high acetic acid production up to 100 mM [22].

The organoid–transwell model is an interesting tool to preclinically assess new therapeutic properties in IBD. Organoid 3D cultures obtained and grown from endoscopic biopsies from patients maintain patient-specific IBD characteristics [23], allowing long-term culturing and the study of intestinal epithelium interactions [24,25]. Here, we studied effects of high acetate (100 mM) on inflammation and intestinal barrier integrity in organoid-derived epithelial monolayer cultures from UC patients to gain further insights on the potential of acetate and thereby also its producing organisms, e.g., *S. boulardii*, in treating IBD.

## 2. Results

### 2.1. Impact of High Acetate Supplementation on Barrier Integrity

**Figure 1:**
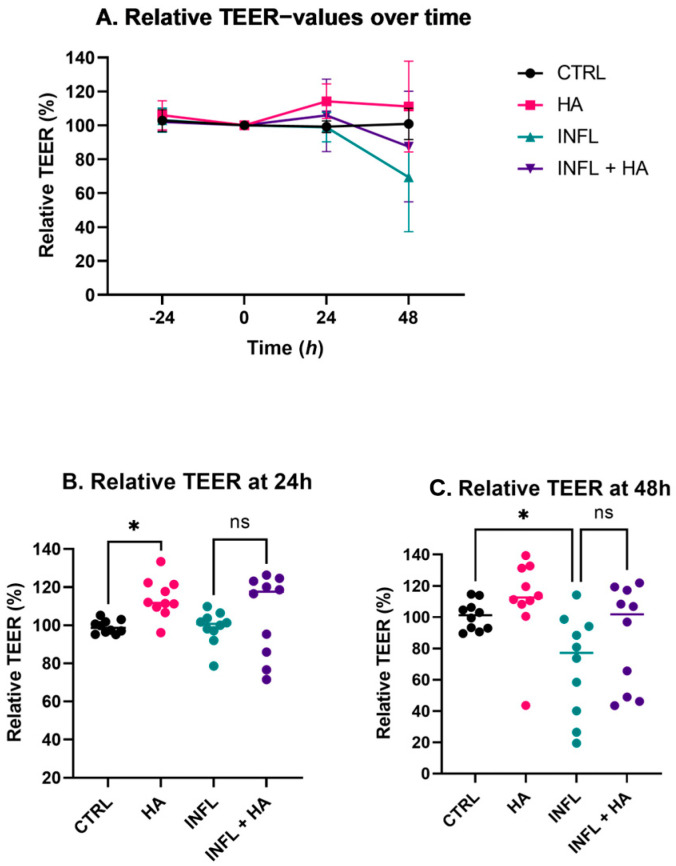
Relative TEER values comparing the mean measurements of duplicate measurements to the mean of each baseline measurement at 0 h. (**A**) Relative TEER over time compared to 0 h (*n* = 10), including means and SD. (**B**) Relative TEER at 24 h (*n* = 10), including results of the Friedman test (* *p* < 0.05; ns = not significant). (**C**) Relative TEER at 48 h (*n* = 10), including results of the Friedman test (* *p* < 0.05; ns = not significant). CTRL = Control medium, INFL = Control medium and inflammatory mix, HA = medium containing 100 mM acetate, INFL + HA = medium containing 100 mM acetate and inflammatory mix.

Upon administration of 100 mM acetate (HA) to the monolayer cultures, a significant increase in TEER compared to the control condition (CTRL) was observed (two-way ANOVA, *adj*. *p* = 0.0047, Figure 1). Moreover, an overall significant difference was noted between the inflamed condition (INFL) and the HA condition without inflammation (two-way ANOVA, *adj*. *p* = 0.0099). At 48 h, basolateral stimulation with inflammatory stimuli Flagellin, IL1B and TNFα led to a numerical though borderline not significant decrease in overall TEER (two-way ANOVA, *adj*. *p* = 0.0557). This decrease in TEER was not observed when high acetate (INFL + HA) was co-incubated (two-way ANOVA, *adj*. *p* = 0.6144, Figure 1A). 

Pairwise comparisons at 24 h between treatment conditions confirmed that HA supplementation in a non-inflamed setting increased TEER values significantly (Friedman, *adj. p* = 0.0487, Figure 1B). In the inflamed conditions, the difference was less prominent, but the same trend was observed (Friedman, *adj. p* = 0.1135, Figure 1B). At 48 h, the same trends were observed (Figure 1C).

### 2.2. High Acetate Administration Downregulates Inflammatory IL 8 and TNFα Expression and Influences Barrier Genes

**Figure 2:**
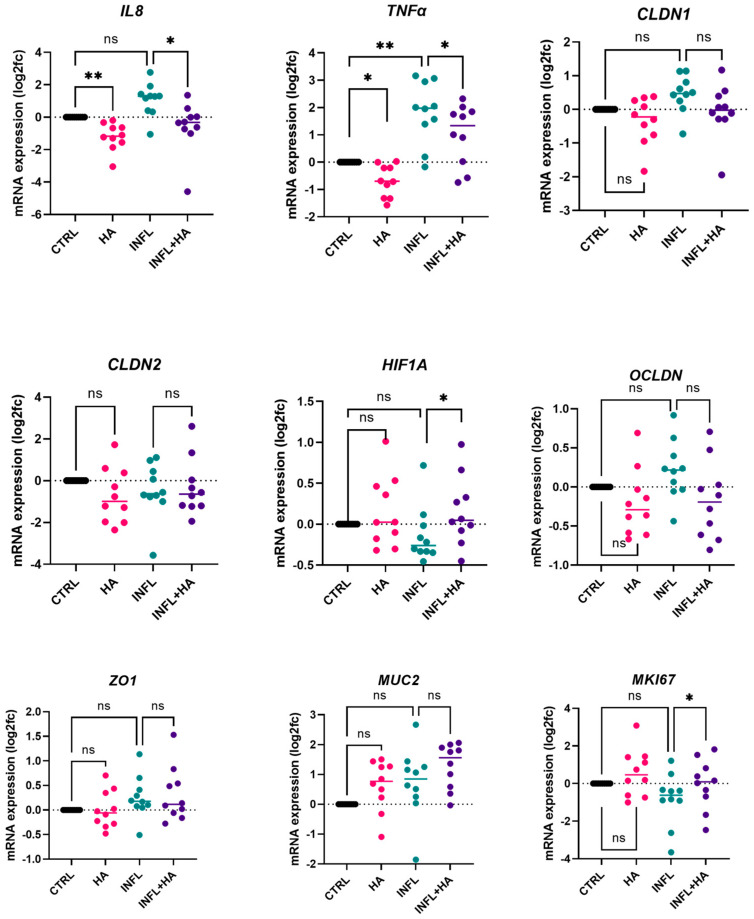
mRNA expression levels at 48 h (*n* = 10) given by log2fold changes (log2fc) compared to the control condition, results of One-way ANOVA or Friedman tests are given (* *p* < 0.05; ** *p* < 0.01; ns = not significant). CTRL = Control medium, INFL = Control medium and inflammatory mix, HA = medium containing 100 mM acetate, INFL + HA = medium containing 100 mM acetate and inflammatory mix.

Following apical acetate stimulation, gene expression levels of IL8 and TNFα were significantly downregulated in both inflamed (IL8: *adj. p* = 0.028; TNFα: *adj. p* = 0.0095) and non-inflamed conditions (Friedman IL8: *adj. p* = 0.0097; One-way ANOVA TNFα: *adj.p* = 0.0095) (Figure 2). 

Next to the evaluation of the TEER, mRNA expression levels of selected barrier genes were analysed by qRT-PCR in the stimulated cultures. Acetate administration resulted in a borderline not significant decrease in CLDN1 expression in the inflamed condition compared to inflammation alone (One-way ANOVA, *adj. p* = 0.084; *non-adj. p* = 0.028) and an increase in CLDN1 expression compared to the CTRL (One-way ANOVA, *adj. p* = 0.077, *non-adj. p* = 0.026). 

HIF1α and MUC2 were respectively significantly and borderline significantly upregulated upon high acetate stimulation in the inflamed condition (HIF1 α: One-way ANOVA, *adj. p* = 0.012; MUC2: One-way ANOVA *adj. p* = 0.061; *non-adj. p* = 0.02). The proliferation marker MKI67 was also significantly upregulated upon high acetate administration (One-way ANOVA *adj. p* = 0.020). 

For the other genes (CLDN2, OCLDN, and ZO1) no (borderline) significant differences or trends were observed. 

### 2.3. Administration of High Acetate Concentrations Decreased Pro-Inflammatory Cytokines in the Apical Transwell Chamber

After exposure to the inflammatory mix and high acetate concentrations, the apical medium was analysed for pro-inflammatory cytokine expression. Pairwise comparisons between the control and inflammatory conditions (Wilcoxon-tests) showed significantly increased concentrations for all proinflammatory cytokines in inflamed conditions compared to the control conditions (all *p* < 0.05), except for IL13 and TNFα (both *p* = 0.065).

**Figure 3:**
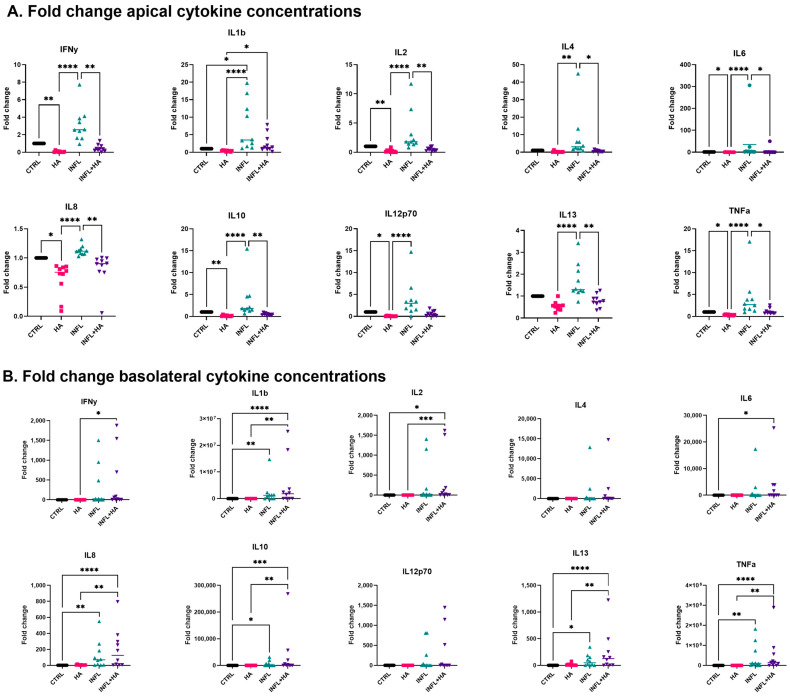
Fold change of proinflammatory cytokine concentrations compared to the respective control, incl. median. All significant and/or clinically relevant p-values (Friedman tests * *p* < 0.05; ** *p* < 0.01, *** *p* < 0.001, **** *p* < 0.0001) have been added to the figure. Values below detection limit were replaced by half the limit of detection (LOD/2). (**A**) Apical medium. (**B**) Basolateral medium. CTRL = Control medium, INFL = Control medium and inflammatory mix, HA = medium containing 100 mM acetate, INFL + HA = medium containing 100 mM acetate and inflammatory mix.

Further, several significant differences in cytokine concentration were noted upon HA stimulation in the apical chamber (Figure 3A). When comparing the concentration fold changes upon HA stimulation in both inflamed and non-inflamed conditions, significant decreases were found for all measured cytokines. Upon Dunn’s correction, results remained significant for IFNγ, IL2, IL6, IL8, IL10, IL13 and TNFα in both inflamed and non-inflamed conditions (Table 1). 

In addition, the basolateral medium was analysed following inflammatory and HA stimulation. The basolateral chamber (Figure 3B) did not show any significant differences upon HA stimulation for non-inflamed subgroups. However, the difference between CTRL and INFL was significant for IL1β (*adj. p* = 0.0081), IL8 (*adj. p* = 0.00360), IL10 (*adj. p* = 0.011), IL13 (*adj. p* = 0.011), and TNFα (*adj. p* = 0.0032). 

## 3. Discussion

In this study, organoid-derived epithelial monolayer cultures from UC patients proved a highly suitable model to assess the effect of HA concentrations on inflammation and intestinal barrier integrity. The organoid model allows the investigation of different mechanisms and responses in a patient-specific manner as cells maintain the characteristics of the original donor [24]. Moreover, easy access to both the apical and basolateral side of the epithelial monolayer is a great advantage of transwell cultures compared to organoid cultures where the lumen is enclosed [25].

The production of HA levels has been suggested as one of the mechanisms driving the probiotic potency of the yeast *Saccharomyces cerevisae* var. *boulardii*. Using the organoid–transwell model, we showed preclinically that HA stimulation indeed protected intestinal barrier integrity in both non-inflamed and inflamed conditions. The presence of inflammation led to a significant decrease in TEER at 48 h while HA stimulation could prevent this reduction, thus suggesting barrier protective properties [18]. This was further confirmed by readouts of CLDN1 which was previously shown to be upregulated in IBD [26,27]. Here, CLDN1 showed increased expression levels upon inflammatory stimulation, while HA stimulation caused a decrease in expression similar to the non-inflamed state. Although CLDN1 has been identified as a tightening claudin, the exact function in IBD remains unclear [26]. Moreover, a complex interplay has been described between CLDN1 and HIF1A [28], an upstream mediator of tight junction function that was significantly upregulated upon HA stimulation in our study. HIF1A has different functions and is thought to be involved in barrier protective mechanisms including production of trefoil factors, mucins, and β-defensins, and the upregulation of mucosal immune responses [29]. Conditional deletion of HIF1A leads to increased susceptibility to colitis in mice [30] and has therefore been extensively linked to beneficial outcomes in murine models of colitis [28]. Collectively, the readouts we obtained for these genes and their functions are thus consistent with the generation of a stronger barrier. 

Next to tight junctions, the mucus layer also plays an important role in intestinal barrier function. Expression levels of MUC2, a marker for the expression of main intestinal mucin, were borderline significantly upregulated upon HA stimulation which may point to a protective effect of the monolayer. Moreover, previous studies showed spontaneous development of colitis in MUC2-deficient mice, which is in line with our findings [31]. Finally, the proliferation marker MKI67 has previously been shown to be downregulated in UC-derived organoids [32]. Yet, upon HA stimulation a significant upregulation of MKI67 in the inflamed condition was observed. The promotion of growth factors including MKI67 might explain the observed protective effect by introducing epithelial repair [33]. Currently, it is still unclear whether these effects on gene expression are direct or indirect consequences of acetate administration, given that similar indirect pathways have been described for butyrate [18].

Furthermore, the anti-inflammatory effects of acetate were evaluated by gene expression levels of IL8 and TNFα which were significantly downregulated upon HA stimulation in both inflamed and non-inflamed conditions showing its anti-inflammatory potency. These anti-inflammatory effects were confirmed by a significant decrease upon HA stimulation in nearly all measured pro-inflammatory cytokines with the exception of IL1b and IL12p70, yet the same trend is present. Moreover, the mechanism of butyrate’s direct beneficial effects has been shown to be histone deacetylase (HDAC) inhibition leading towards increased expression levels of HIF1, STAT3, and SP1, as well as decreased expression of NFkB [18]. Based on our results, we suggest similar acetate-induced HDAC inhibition. Remarkably, the anti-inflammatory cytokine IL10 was significantly decreased upon HA stimulation. This observation was unexpected as butyrate has previously been shown to increase IL10 concentration upon LPS stimulation [34]. However, this shows that there are differences between butyrate and acetate supplementation.

So far, butyrate supplementation has been studied more intensively compared to acetate. Previously, Vancamelbeke et al. (2019) showed that butyrate supplementation at physiological concentrations of 8 mM was detrimental in inflamed conditions. It is worth noting that while the same experimental set-up was used as in this study [35], another inflammatory mix was used in our experiments [25]. Yet, this effect was not observed in our experiments upon stimulation with 100 mM acetate, which is estimated to be four times higher than physiological concentrations in the lumen of the gut [18,35]. This confirms that butyrate might be more detrimental or toxic [21] to the epithelial barrier compared to acetate.

As very limited data are available on absolute cytokine levels in organoid-derived cell culture media [35], clinical interpretation of minor differences observed in cytokine concentrations is challenging and merits further discussion. Therefore, fold changes are used to compare conditions to the control. Most clinical data have been published on serum or plasma cytokine levels, for which concentrations are usually higher than measured in cell culture media [36,37]. However, as the surface of the transwell is limited, in vivo stimulations might lead to higher and clinically relevant differences, though this remains to be elucidated. Yet, a relative difference was observed in this study, which tends to be a positive signal.

A limitation of our transwell model concerns the lack of immune cells and of gut microbiome interactions characteristic for the in vivo situation [36,37]. So far, the addition of biologically relevant stimuli such as gut metabolites to our current setup only allows us to study effects on epithelial cells, whereas multiple other interactions might also take place. Butyrate, for example, has also been shown to exert beneficial effects on macrophages and T cells [18]. It is not unlikely that acetate might also positively contribute to this process through metabolic cross-feeding interaction with acetate-assimilating butyrate producers [18]. Therefore, follow-up in vitro micro-fermentation experiments as well as in vivo studies are mandatory to fully elucidate the effects of HA concentrations.

Our results suggest that HA administration might be of benefit in barrier-defected and inflammatory diseases such as UC, but also other disorders such as travellers’ diarrhoea. However, administration of pure acetate to patients should be considered cautiously considering that partial or complete metabolization before reaching the colon might severely compromise treatment efficiency. On the other hand, our results support the hypothesis that the unusually high production of acetic acid by the probiotic yeast *S. cerevisae* var. *boulardii*, as opposed to the closely related yeast *S. cerevisiae*, might be responsible for its probiotic potency [22]. Therefore, natural or engineered probiotics that can locally produce HA concentrations might be the way forward to support IBD management.

## 4. Materials and Methods

The protocol was based on Van Dussen et al. (2015), Vancamelbeke et al. (2019), and Arnauts et al. (2020) [25,35,38].

### 4.1. Human Biopsy Collection and Ethical Statement

Mucosal biopsies from macroscopically non-inflamed colon tissue were obtained during routine endoscopy from 10 UC patients following informed consent (S53684; approved by the Ethics Committee of the University Hospitals Leuven). The baseline characteristics of these patients are given in Table 2.

Biopsies were collected in ice-cold basal medium (BM) (Appendix A) (DMEM/F12 1:1 Mixture (Lonza, Basel, Switzerland) supplemented with 1× GlutaMAX [Gibco, Thermo Fisher Scientific, Waltham, MA, USA), 10 mM HEPES (Gibco, Thermo Fisher Scientific, Waltham, MA, USA), and 100 μg/mL penicillin/streptomycin (Gibco, Thermo Fisher Scientific, Waltham, MA, USA)) and processed within 2 h for crypt isolation. 

### 4.2. Intestinal Crypt Isolation and Organoid Culture

Intestinal crypts were isolated from 4–6 biopsies per patients as previously described by VanDussen et al. (2015). Following isolation, crypts were resuspended in Matrigel (Growth Factor Reduced, phenol-red-free, Corning, NY, USA) and diluted with basal medium (50:50). Four droplets (10 μL) of this suspension were plated on every well of a 24-well tissue culture plate; including 8–12 wells/4–6 biopsies, depending on the biopsy size and isolation efficiency. Next, the culture plates were incubated at 37 °C and 5% CO_2_ for at least 20 min, which enables polymerisation of the Matrigel. Next, human expansion medium (HM) (Appendix B) was added to the wells to expand the organoids. The medium was replaced every 48 h, and the organoids were split after 7–10 days, usually 1:3, depending on their growth rate.

Every new culture was registered and several vials of at least 3 wells with a low passage number (P2–P4) were pelleted and resuspended in 700 µL Recovery Cell Culture Freezing Medium (Gibco, Thermo Fisher Scientific, Waltham, MA, USA) and stored in liquid nitrogen until further use.

### 4.3. Primary Epithelial Monolayer Cultures

Before each monolayer experiment, one sample aliquot containing colonic organoids was slowly thawed, resuspended in Matrigel mixture, and expanded until enough wells were obtained. Next, 6.5 mm transwell inserts (CLS3470, 0.4-μm pore PET membrane, Corning Costar) were coated with 0.1 mg/mL collagen type I (rat tail, Corning) diluted in 0.2 M acetic acid overnight at 37 °C. The next day, the transwells were rinsed three times with phosphate buffered saline (PBS) (Gibco, Thermo Fisher Scientific, Waltham, MA, USA) and pre-incubated with 50% HM, diluted with BM, and 10 μM Rho-associated kinase (ROCK) inhibitor (Y-27632, Selleckchem, Munich, Germany). For every transwell insert, approximately 3 wells with organoids were harvested 3–4 days after last splitting and between passage numbers 5 and 10 in total. This was followed by mechanical dissociation by pipetting up and down.

Next, organoids were washed in 0.5 mM EDTA/PBS solution and centrifuged for 5 minutes (min) at 350× *g*. The pellets were next treated with 0.25% trypsin/EDTA (Gibco, Thermo Fisher Scientific, Waltham, MA, USA) for 5 min at 37 °C in a water bath and further mechanically dissociated by pipetting. During this dissociation process, cells were regularly checked under a standard light microscope until a homogeneous solution without visible aggregates is obtained. Next, trypsin was inactivated using an excess of BM supplemented with 10% foetal bovine serum. Cells were spun down at 350× *g* for 5 min. Finally, cells were dissolved in 900 µL of 50% HM + ROCK inhibitor to seed 100 µL in each apical compartment (*n* = 8). The basolateral compartment was filled with 600 μL of 50% HM + ROCK inhibitor. 

To let cells attach and grow, they were kept at 37 °C in a humidified incubator with 5% CO_2_. After 24 h, dead and un-attached cells were washed away by carefully pipetting up and down without touching the membrane. The medium in the apical (200 μL) and basolateral compartments (600 μL) was refreshed with 50% HM without ROCK inhibitor, and this was repeated every other day until confluent and polarised monolayers were formed.

### 4.4. Induction of Inflammation and High Acetate Treatment

Confluency of the monolayer was evaluated by transepithelial electrical resistance (TEER) measurement. When confluency and polarisation were reached (usually 5–7 days after seeding in the transwells), cells were basolaterally stimulated with control medium (CTRL) or an inflammatory mix [25] (INFL) containing 100 ng/mL TNF-α, 20 ng/mL IL1β and 1 µg/mL Flagellin. After 24 h, cells were apically stimulated with control medium (50% HM) or high acetate (HA): 100 mM sodium acetate (Sigma-Aldrich, St. Louis, MO, USA; Merck, Rahway, NJ, USA) dissolved in 50% HM. All conditions were tested in duplicate. TEER was measured at 0, 24, and 48 h of stimulation. After 48 h, cells were washed once with PBS. Next, cultures were incubated for five minutes with 0.25% Trypsin–EDTA. The monolayers were mechanically dissociated and collected with BM supplemented with 10% FBS to stop the trypsinisation. The pellet was washed once with PBS before addition of lysis buffer and 12 mM β-mercapto-ethanol. RNA-extraction was performed using the Promega ReliaPrep™ miRNA Cell and Tissue Miniprep System. RNA extraction was followed by reverse transcriptase qPCR targeting a selection of key diagnostic marker genes (primers in Appendix C). The apical and basolateral media were collected for cytokine determination by using the Meso Scale Discovery platform as described below.

### 4.5. Transepithelial Electrical Resistance Measurements

TEER measurements were performed using an EVOM epithelial Volt/Ohm meter and STX2 chopstick electrode set (World Precision Instruments, Sarasota, FL, USA). All measurements were performed in duplicate. Final values were calculated as relative values which compare the mean measurements per condition to the mean of each baseline measurement at 0 h.

### 4.6. Gene Expression Analysis by Quantitative Reverse Transcription

Cells were immersed in RNA lysis buffer (Qiagen, Hilden, Germany) with 2-mercaptoethanol (Sigma-Aldrich) at isolation, and lysates were kept at −80 °C until RNA extraction. RNA was isolated by using the ReliaPrep miRNA Cell and Tissue Miniprep System (Promega, Fitchburg, WI, USA) according to the standardized protocol by the company. Complementary DNA was synthesised using the SuperScript III First-Strand Synthesis SuperMix for qRT-PCR (Invitrogen, Waltham, MA, USA) according to the manufacturer’s protocol. 

The expression of major genes involved in intestinal barrier integrity were studied: claudin 1 (CLDN1), claudin 2 (CLDN2), occludin (OCLN), zonula occludens 1 (ZO1), and mucin 2 (MUC2). Hypoxia-inducible factor 1A (HIF1A) was also measured as an upstream mediator of tight junction function. Interleukin-8 (IL8) and Tumor Necrosis Factor α (TNF-α) were added as inflammatory markers, and marker of proliferation Ki-67 (MKI67) was quantified. Primers (Appendix C) were designed using OligoAnalyzer 3.1 software (Integrated DNA technologies, Coralville, IA, USA) or Primer-BLAST. All reactions were performed in duplicate on a Lightcycler 96 PCR machine (Roche, Basel, Switzerland). Results are presented as log2 fold change, relative to the mean of endogenous reference genes: ribosomal protein S14 (RPS14), beta-2-microglobulin (B2M), and beta-actin (ACTB).

### 4.7. Cytokine Profiling

Cytokine levels were quantified in apical and basolateral media using an electro-chemi-luminescence-based Meso Scale Discovery (MSD) platform exploiting the V-PLEX Proinflammatory Panel 1 Human Kit. This kit provides assay-specific components for the quantitative determination of IFNγ, IL1β, IL2, IL4, IL6, IL8, IL10, IL12p70, IL13, and TNFα. Preparation of samples and detection plates were performed following the manufacturer’s instructions (Meso Scale Diagnostics, Rockville, MD, USA) with few adaptations. In short, a series of 9 concentrations of standards in duplo, together with the samples were added to the plate. Plates were incubated while shaking at room temperature for 2 h. After incubation, plates were washed twice, and the respective detection antibody mixture was added to each well. Again, plates were incubated while shaking at room temperature for 2 h. Afterwards, plates were washed and 2X Read buffer was added to each well. Plates were read on the MSD Plate reader (MESO QuickPlex SQ 120, Meso Scale Diagnostics, Rockville, MD, USA). Protein concentrations were determined using the MSD Discovery Workbench 4.0 analysis software. Results are presented as fold change compared to the control for each culture. 

### 4.8. Statistical Analysis

Data were analysed using GraphPad Prism 9 (San Diego, CA, USA). Continuous data that were normally distributed (D’Agostino and Pearson test followed by q-q plot analysis and Levene’s test for equal variances) were presented as mean and standard deviation (SD). Data not normally distributed were presented as median with interquartile (IQR) ranges. Comparisons between treatment groups and time-dependent analyses of TEER, gene expression levels, and protein markers were performed using RM one-way ANOVA followed by Bonferroni correction or paired Friedman tests followed by post-hoc Dunn’s tests. 

## 5. Patents

VIB and KU Leuven have submitted patent applications (15 September 2017; EP 17191252.0. and 27 January 2022; EP 22153700.4.) based on the possible beneficial effect of *S. boulardii*-produced acetic acid for its commercial use as a probiotic.

## Figures and Tables

**Table 1:** *p*-values are given as the result of a Friedman test followed by post-hoc Dunn’s test without correction (*p*) and with correction (*adj. p*) comparing the mean cytokine concentrations of 10 biological repeats. CTRL = Control medium, INFL = Control medium and inflammatory mix, HA = medium containing 100 mM acetate, INFL + HA = medium containing 100 mM acetate and inflammatory mix.

Cytokine		CTRL vs. HA	CTRL vs. INFL	CTRL vs. INFL + HA	HA vs. INFL	HA vs. INFL + HA	INFL vs. INFL + HA
IFNy	*p*	0.0007	0.119	0.0999	<0.0001	0.0833	0.0014
	*Adj. p*	0.0044	0.7142	0.5993	<0.0001	0.4996	0.0081
IL1b	*p*	0.0153	0.0056	0.729	<0.0001	0.0056	0.0153
	*Adj. p*	0.0919	0.0335	>0.9999	<0.0001	0.0335	0.0919
IL2	*p*	0.001	0.119	0.0833	<0.0001	0.119	0.001
	*Adj. p*	0.006	0.7142	0.4996	<0.0001	0.7142	0.006
IL4	*p*	0.0377	0.119	0.119	0.0003	0.6033	0.0018
	*Adj. p*	0.226	0.7142	0.7142	0.0017	>0.9999	0.0109
IL6	*p*	0.0073	0.0464	0.2987	<0.0001	0.0999	0.0024
	*Adj. p*	0.0436	0.2783	>0.9999	<0.0001	0.5993	0.0146
IL8	*p*	0.0018	0.0567	0.119	<0.0001	0.119	0.0005
	*Adj. p*	0.0109	0.3405	0.7142	<0.0001	0.7142	0.0032
IL10	*p*	0.0003	0.1659	0.0567	<0.0001	0.0833	0.001
	*Adj. p*	0.0017	0.9951	0.3405	<0.0001	0.4996	0.006
IL12p70	*p*	0.0032	0.119	0.4884	<0.0001	0.0243	0.0243
	*Adj. p*	0.0194	0.7142	>0.9999	<0.0001	0.1461	0.1461
IL13	*p*	0.0094	0.0567	0.1659	<0.0001	0.2253	0.001
	*Adj. p*	0.0562	0.3405	0.9951	<0.0001	>0.9999	0.006
TNFα	*p*	0.0018	0.0567	0.3865	<0.0001	0.0243	0.0056
	*Adj. p*	0.0109	0.3405	>0.9999	<0.0001	0.1461	0.0335

**Table 2:** Baseline characteristics of patients (*n* = 10) at inclusion. IQR = interquartile range.

Male/female [%]	5/5 [50%]
Age at inclusion in years: Median [IQR]	47.5 [10.25]
Disease duration in years: Median [IQR]	12.5 [5.25]
Total Mayo score: Median [IQR]	8.5 [3.75]
Endoscopic Mayo subscore: [%]	
2	5 [50%]
3	5 [50%]
Medication use [%]	7 [70%]
5-aminosalicylates	4 [40%]
Corticosteroids	1 [10%]
Immunomodulator	0 [0%]
Biologicals	3 [30%]
Small molecules	0 [0%]

## Data Availability

The data presented in this study are available on request from the corresponding author.

## Appendix A. Composition Basal Medium (BM)

Basal medium [BM]:

- DMEM/F12 1:1 Mixture (Lonza, Basel, Switzerland) supplemented with
- 1× GlutaMAX (Gibco, Thermo Fisher Scientific, Waltham, MA, USA),
- 10 mM HEPES (Gibco, Thermo Fisher Scientific, Waltham, MA, USA), and
- 100 μg/mL penicillin/streptomycin (Gibco, Thermo Fisher Scientific, Waltham, MA, USA).

## Appendix B. Composition Human Expansion Medium (HM)—*v*/*v*: Percentage Volume of Total End Volume

**Component**	**Concentration**	**Manufacturer**	**Catalog nr**
Wnt3A	50% *v/v*	In house cell line	/
R-spondin	20% *v/v*	In house cell line	/
Noggin	10% *v/v*	In house cell line	/
EGF	50 ng/mL	Life Technologies	PMG8041
A83-01	500 nM	Tocris	2939/10
SB202190	10 µM	Sigma-Aldrich	S7067
Nicotinamide	10 mM	Sigma-Aldrich	N0636
n-Acetylcysteine	1.25 mM	Sigma-Aldrich	A9165
B27	1×	Life Technologies	17504044

## Appendix C. Sequence Primers qPCR

**Name**	**Sequence**	**Scale**	**Purification**
ACTB forward	CCCAGCACAATGAAGATCAAGATC	25 nm	STD
ACTB reverse	CTGATCCACATCTGCTGGAAG	25 nm	STD
B2M forward	TGCTGTCTCCATGTTTGATGTATCT	25 nm	STD
B2M reverse	TCTCTGCTCCCCACCTCTAAGT	25 nm	STD
CLDN1 forward	GCAGATCCAGTGCAAAGTC	25 nm	STD
CLDN1 reverse	CTATCACTCCCAGGAGGATG	25 nm	STD
CLDN2 forward	CACACTGGTTGCCATGCT	25 nm	STD
CLDN2 reverse	ATTCCATCCAGAGGCCCT	25 nm	STD
HIF1A forward	CTAACTAGCCGAGGAAGAACTATGA	25 nm	STD
HIF1A reverse	TGGTTACTGTTGGTATCATATACGTG	25 nm	STD
IL8 forward	ACTGAGAGTGATTGAGAGTGGAC	25 nm	STD
IL8 reverse	AACCCTCTGCACCCAGTTTTC	25 nm	STD
MKI67 forward	AAAGGCAAAGAAGACCTGCTA	25 nm	STD
MKI67 reverse	TTTGCGTGGCCTGTACTAAAT	25 nm	STD
MUC2 forward	CACCAAGACCGTCCTCATG	25 nm	STD
MUC2 reverse	CTTGGCCGAGTACATGACA	25 nm	STD
OCLN forward	TGGCAAAGTGAATGACAAGC	25 nm	STD
OCLN reverse	AGGCGAAGTTAATGGAAGCTC	25 nm	STD
RPS14 forward	TCACCGCCCTACACATCAAAC	25 nm	STD
RPS14 reverse	GCCCGATCTTCATACCCGA	25 nm	STD
TNFA forward	TAGCCCATGTTGTAGCAAACCC	25 nm	STD
TNFA reverse	TATCTCTCAGCTCCACGCCA	25 nm	STD
ZO1 forward	AAAGAAGCAATTCAACAACAGCAA	25 nm	STD
ZO1 reverse	ATCATGCAAATCAAGGTCATCACT	25 nm	STD
